# Efficacy of a novel surgical approach: sutureless correction of conjunctivochalasis using new conjunctival forceps combined with high-frequency electrocautery

**DOI:** 10.3389/fopht.2025.1554316

**Published:** 2025-07-11

**Authors:** Tingting Song, Xianjun Liang, Yingjie Lin, Huihui Luo

**Affiliations:** ^1^ Department of Ophthalmology, Shijingshan Teaching Hospital of Capital Medical University, Beijing Shijingshan Hospital, Beijing, China; ^2^ Foshan Aier Zhuoyue Eye Hospital, Foshan, Guangdong, China

**Keywords:** conjunctivochalasis, new conjunctival forceps, high-frequency electrocautery, lip-parallel conjunctival folds, sutureless

## Abstract

**Purpose:**

To investigate the efficiency, efficacy, and safety of application new conjunctival forceps combined with high-frequency electrocautery-assisted for treatment of conjunctivochalasis (Cch).

**Methods:**

This study included data from 19 patients (22 eyes)with conjunctivochalasis who underwent treatment from January to December 2023. Cch correction was performed with our new conjunctival forceps (Patent Number: ZL 2022 2 0320601.0), which offers enhanced precision and stability in grasping the conjunctiva, combined with high-frequency electrocautery (Ellman, America) for sutureless correction. Surgical duration was recorded. Cch severity, assessed with lip-parallel conjunctival folds (LIPCOF), along with discomfort scored by Ocular Surface Disease Index (OSDI), were compared pre- and post-operatively. A reduction in LIPCOF grade and complete healing of the conjunctival epithelium post-surgery within one month were considered successful outcomes. Healing and potential complications were checked at one week and one month.

**Results:**

The mean surgical duration was 6.9 ± 1.5 minutes. Significant improvement in LIPCOF grades was observed over time (χ²=62.824, p<0.01), with 59.1% of eyes achieving grade 0 at 1 week postoperatively, increasing to 95.5% at 1 month. OSDI scores showed no difference between preoperative (38.54) and 1-week postoperative (41.67) values (p=0.922), but significantly decreased by 1 month (16.67, p<0.001). Complete healing was noted at electrode contact sites by 1 week, while incisions managed with novel conjunctival forceps showed 75% healing rate; all incisions were fully healed by 1 month. Mild postoperative congestion and edema resolved completely without major complications.

**Conclusion:**

The new conjunctival forceps with high-frequency electrocautery for sutureless Cch correction proved to be efficient, effective, and safe.

## 1 Introduction

Conjunctivochalasis (Cch) is a chronic conjunctival disorder characterized by non-edematous and lax conjunctival folds (1). It is more commonly observed in the bulbar conjunctiva of the lower eyelid and often leads to tear film dysfunction. Although the exact cause of Cch is unknown, aging is linked to the condition. Additionally, studies indicate that aberrant elastic fiber formation and progression are pathogenic traits (2). In contemporary life, a high incidence of dry eye syndrome has led to gradual youthfulness in the population affected by Cch and can even exacerbate the condition of dry eye syndrome related to meibomian gland dysfunction (3).Varying severities of conjunctivochalasis (Cch) can lead to a spectrum of symptoms; lesser cases may provide a sense of a foreign body, while more severe cases may cause burning, redness in the eyes, tears, and keratoconjunctivitis, among other symptoms. In the early stages of Cch, treatment often involves symptomatic relief, such as the use of artificial tears (4), and treatments include topical medication and laser therapy (5, 6). If conservative treatment is ineffective, then surgical intervention is necessary (3, 5). Traditional surgical methods for the treatment of Cch involve conjunctival resection and, when necessary, amniotic membrane transplantation (AMT) (7). These procedures necessitate sutures and their later removal, a process that is both time-consuming and cumbersome. In recent years, some scholars have performed conjunctival correction assisted by high-frequency electrocautery (3), which is simple and time-saving, but the correction for Cch above grade II is limited. In order to accomplish a quick and easy sutureless Cch correction, this study presents a novel technique that combines high-frequency electrocautery with conjunctival forceps.

To overcome the aforementioned limitations, this study presents an innovative surgical technique for the correction of conjunctivochalasis (Cch), which integrates a patented conjunctival forceps with high-frequency electrocautery. This novel approach is designed to optimize the surgical workflow and improve clinical outcomes. The patented forceps incorporate a spring-lock mechanism that securely immobilizes tissue, thereby eliminating the need for an assistant to maintain manual traction and enabling the surgeon to operate with enhanced precision and efficiency. Furthermore, the forceps are engineered with a unique head-and-body structure; the head features a tapered, thick-to-thin toothed design that provides a larger and more reliable grasping surface for tissue manipulation. This anatomically inspired design facilitates superior control and stability during surgical procedures, particularly in the context of Cch correction.

The novel technique represents a significant departure from traditional surgical methods. Unlike conventional approaches that focus on the removal of excess conjunctival tissue and subsequent suturing, which can be time-consuming and lead to complications such as symblepharon formation, this new method emphasizes the restoration of the anatomical and functional integrity of the ocular surface. By integrating the precision of electrocautery with the enhanced grasping capabilities of the patented forceps, the surgical procedure is significantly shortened, and the risk of complications is reduced. This study aims to demonstrate the clinical application and potential benefits of this innovative surgical technique in the correction of conjunctivochalasis, highlighting its potential to improve patient outcomes and enhance surgical efficiency.

## 2 Methods

### 2.1 New conjunctival forceps

**Figure 1:**
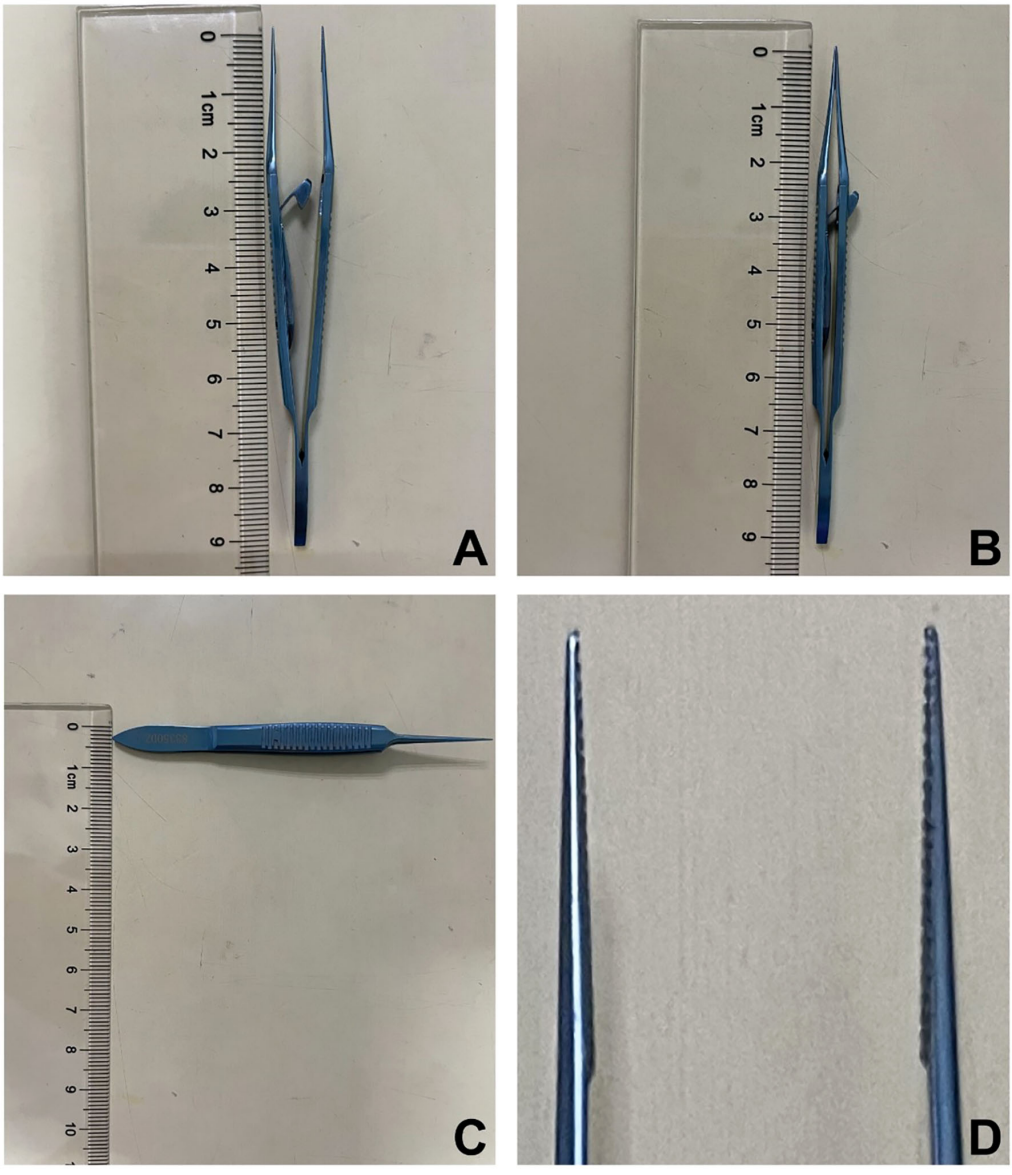
**(A)** Open position of the new conjunctival forceps; **(B)** Closed position of the new conjunctival forceps; **(C)** Width of the new conjunctival forceps; **(D)** The head of the new conjunctival forceps features a serrated matching that tapers from thick to thin.

The new conjunctival forceps are constructed from high-grade titanium alloy, ensuring both durability and biocompatibility. The design consists of two main components: the head and the body. The head, measuring 9.2 cm in length and 0.5 cm in wide, features a serrated edge that tapers from thick to thin. This design is specifically engineered to provide a secure and stable grip on the bulbar conjunctiva, allowing for precise tissue excision while minimizing residual tissue. The body is equipped with a spring-loaded latch mechanism. Once the head clamps onto the bulbar conjunctiva, the spring-loaded latch can be engaged with a simple press, securing the tissue in place. This feature enables the surgeon to independently complete the procedure without requiring an assistant to manually hold the tissue, thereby enhancing surgical efficiency and precision. (**Figure 1** illustrates the detailed structure and mechanism of the new conjunctival forceps, highlighting its innovative design and functional advantages over existing tools).

### 2.2 Patients

Among the 19 patients who visited Foshan Aier Zhuoyue Eye Hospital between January 2023 and December 2023, 22 eyes were given the diagnosis of “Cch”. Inclusion criteria: Individuals with slit-lamp examination-diagnosed Cch who had not responded to conservative treatment for more than three months, including artificial tears and low-concentration corticosteroids. Patients with uncontrolled systemic diseases, implanted cardiac pacemakers or stents, or acute ocular emergencies were excluded. After routine examination, patients underwent Cch correction assisted by new conjunctival forceps combined with high-frequency electrocautery. This study adhered to the Declaration of Helsinki and was approved by the Ethics Committee of the Foshan Aier Zhuoyue Eye Hospital (approval number: 2023IRB2). All the patients provided informed consent to participate in the study.

### 2.3 Surgical procedure

The surgery was performed by the same surgeon. Supine was the posture in which the patients were positioned. Following a standard cleansing of the skin and the edges of the eyelids with 0.5% An’erdian (Type III), the eyelids were retracted using a speculum. Instilling 0.5% Alcaine eye drops (Alcon, USA) for topical anesthesia, medical sodium hyaluronate gel (Bausch & Lomb Incorporated) to protect the cornea, and using high-frequency electrocautery (Ellman Surgitron; Ellman International, Inc., Hewlett, NY), set to coagulation mode with an energy setting of 1, and select a wide tip electrode (8).

**Figure 2:**
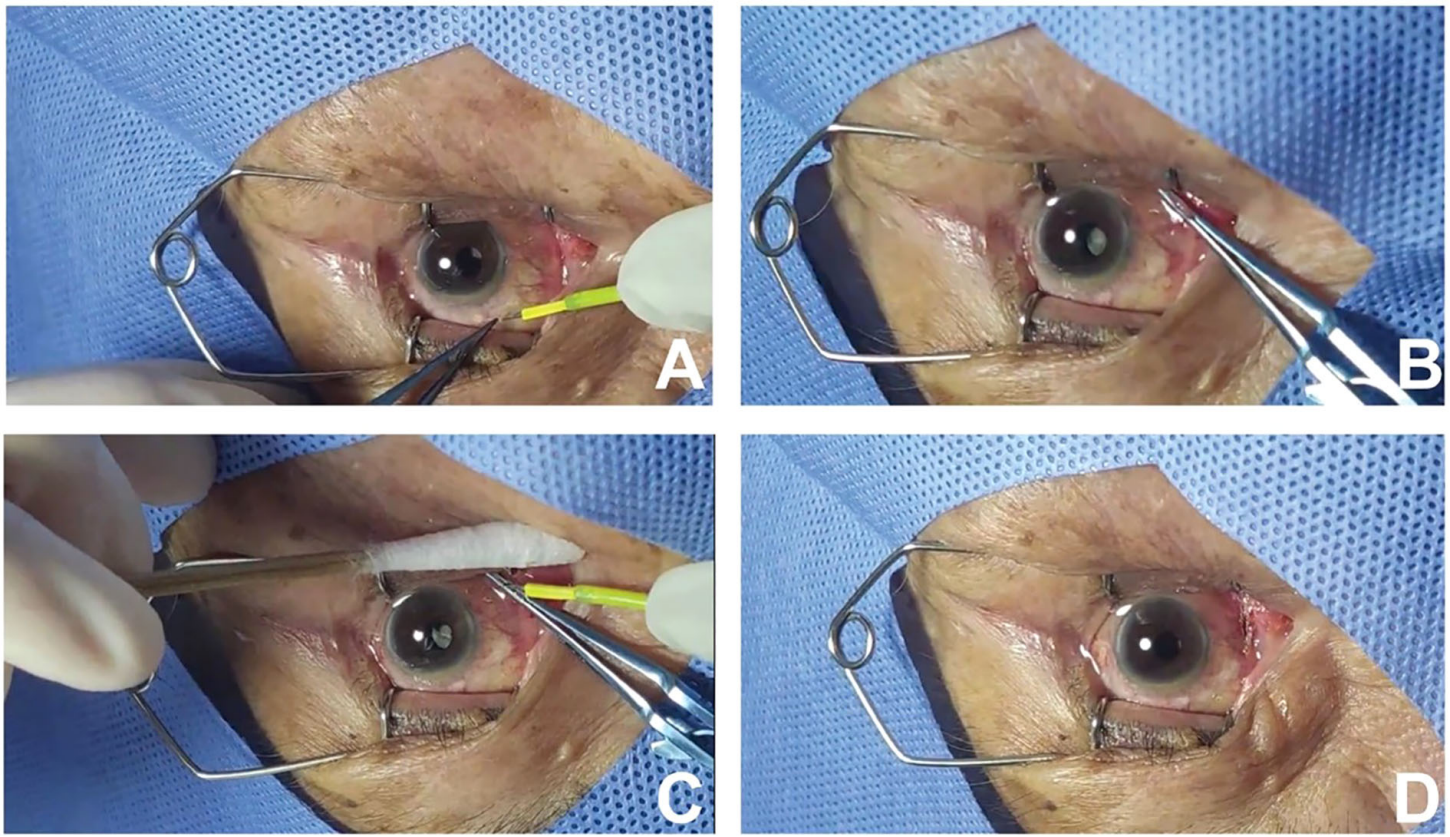
**(A)** If the height of the lifted bulbar conjunctiva does not exceed 3 mm, the tissue is touched several times with the electrode tip. **(B)** Mounting the intended conjunctival tissue for resection with the new conjunctival forceps. **(C)** After excising the lax conjunctiva with microscissors, leaving a residual of 0.1 to 0.2mm at the base, cauterize to seal the incision with the electrode tip. **(D)** The incision is neat after the new conjunctival forceps are released. .

At a distance of 3–5 mm behind the limbus, microforces were used to lift the bulbar conjunctiva in a clockwise direction at each clock-hour position. The decision to use either shrinking or excision was based on the height of the conjunctiva lifted:

If the height lifted did not exceed 3 mm, the lifted conjunctival tissue was touched several times with the electrode tip until satisfactory shrinkage of the conjunctival tissue was achieved. (**Figure 2A**).

If the height lifted exceeds 3 mm (commonly seen in the conjunctival folds and the temporal side of the bulbar conjunctiva), Then perform infiltration anesthesia by injecting 0.1 ml of 0.2% lidocaine solution subconjunctivally with a 30-gauge needle (**Figure 2B**). A minimal amount of anesthesia is applied, which has a negligible impact on the inherent pathological anatomy of the lax conjunctiva. Subsequently, clamp the intended conjunctival tissue for resection with the new conjunctival forceps, excise with microscissors, leaving a residual of 0.1 to 0.2 mm at the base, and then cauterize to seal the incision with the electrode tip. (**Figures 2C, D**).

After surgery, the operated eye was treated with an ophthalmic suspension of tobramycin and dexamethasone (Sa. Alcon-Couvreur N.V.) four times a day for one week and an ophthalmic solution of polyethylene glycol (Alcon Laboratories, Inc.) four times a day for one month. In the second week, the ophthalmic suspension of tobramycin and dexamethasone was switched to an ophthalmic suspension of loteprednol etabonate (Bausch & Lomb Incorporated) four times a day for three weeks. All patients underwent follow-up assessments at 1 week and 1 month postoperatively.

### 2.4 Surgical evaluation

#### 2.4.1 Duration of surgery

The surgical time was reported by the same nurse using a clock, beginning when the eye-speculum was opened and ending when the eye-speculum was removed.

#### 2.4.2 Degree of Cch

With slit-lamp examination, the degree of Cch was classified according to the lid-parallel conjunctival folds (LIPCOF) classification system (grade 0: no conjunctival folds; grade I: a single small fold; Grade II: More twice but not exceeding the height of the tear meniscus; Grade III: Multiple folds that exceed the height of the tear meniscus) (3).

#### 2.4.3 Ocular surface disease index assessment

The Ocular Surface Disease Index (OSDI; Allergan, Irvine, CA, USA), a validated 12-item instrument for assessing dry eye symptoms and their vision-related quality of life impact, was employed. OSDI evaluations were conducted preoperatively, at 1-week postoperative, and at 1-month postoperative intervals to document the temporal pattern of symptomatic improvement after surgical intervention.

#### 2.4.4 Wound healing assessment by fluorescein staining

Fluorescein staining with slit-lamp examination served as the primary wound assessment method, utilizing its high sensitivity in epithelial defect detection. Wound healing status was determined by the absence (healed) or presence (unhealed) of stain uptake at the incision site.

### 2.5 Statistics

All statistical analyses were performed using IBM SPSS Statistics software (version 29.0; IBM Corp., Armonk, NY, USA). Non-normally distributed OSDI scores (confirmed by Kolmogorov-Smirnov/Shapiro-Wilk tests, both p<0.01) were analyzed using Kruskal-Wallis test (H=38.02, p<0.001) with Dunn-Bonferroni *post-hoc* comparisons showing significant differences between all groups (p<0.001) except Group 1.0 vs 2.0 (p=0.922). For LIPCOF grades, chi-square tests revealed significant temporal variations (χ²=62.82, p<0.01), with pairwise comparisons demonstrating: preoperative vs 1-week (χ²=27.27, p<0.001), preoperative vs 1-month (χ²=36.00, p<0.001), and non-significant 1-week vs 1-month differences (χ²=8.73, p=0.101). All tests were two-tailed (α=0.05), with continuous variables presented as mean ± standard deviation, or median (IQR) and categorical data as percentages.

## 3 Results

A total of 19 patients with 22 eyes diagnosed with Cch were included in this study; 7 of the patients were men and 12 were women, with an average age of 68.8 ± 10.3 years.

### 3.1 Surgical time

The average surgery time was (6.9 ± 1.5) minutes.

### 3.2 LIPCOF grades

**Figure 3:**
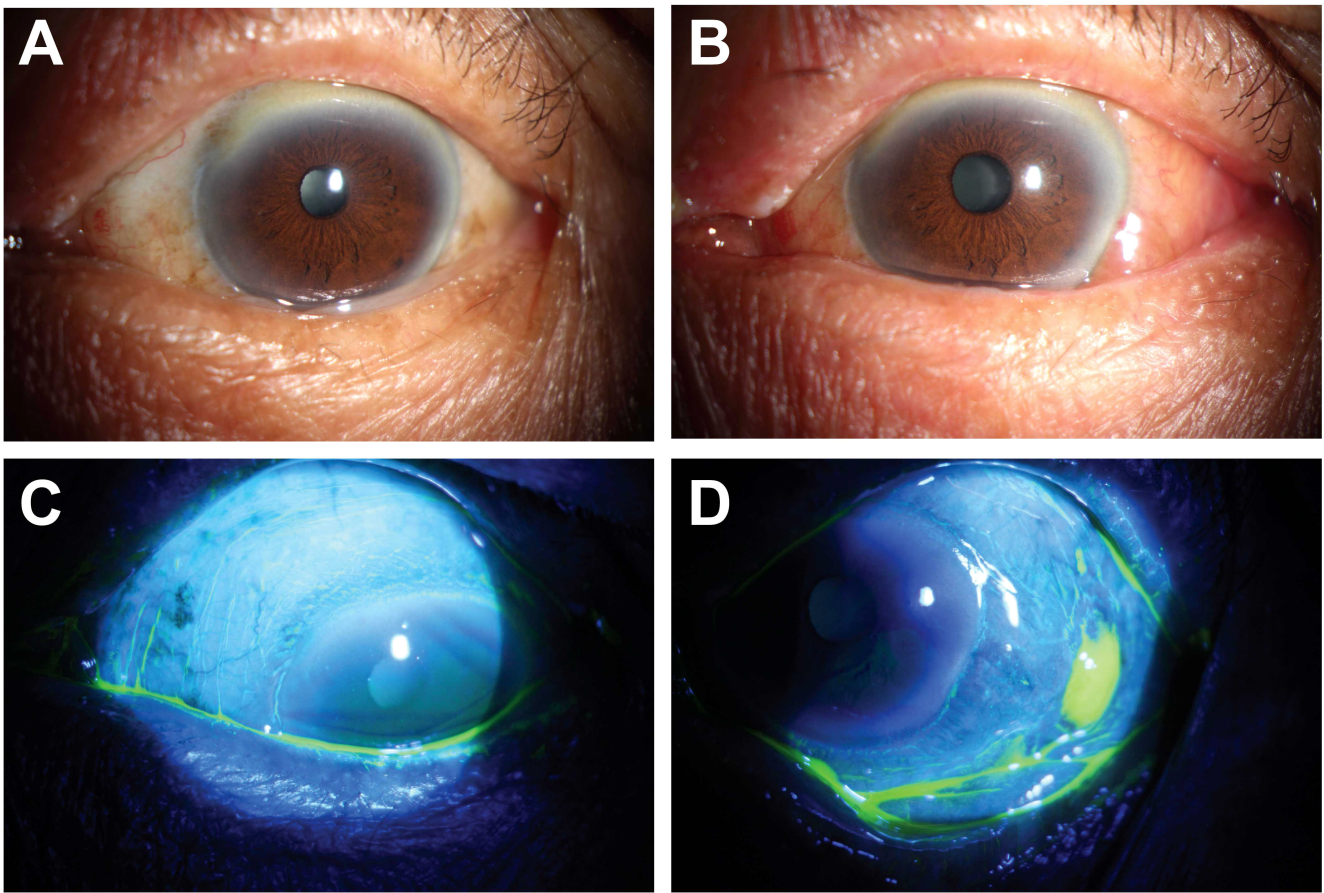
Preoperative and 1-week postoperative external eye photographs of a patient with grade III CCh. **(A)** shows preoperative bulbar conjunctival folds that exceeded the height of the tear meniscus; **(B)** shows 1 week later that the CCh had been corrected, but there was still mild to moderate congestion and edema; **(C)** indicates complete healing of the area where the electrode tip contacted and shrunk the conjunctiva; **(D)** indicates the incision where the new conjunctival forceps clamped, resected and the electrocautery sealed was not fully healed.

The LIPCOF grades of each patient before and after surgery are shown in the **Table 1** below. (**Table 1**, **Figure 3**) Statistical analysis using the chi-square test demonstrated significant temporal variations in LIPCOF grade distribution across three timepoints (preoperative, 1-week postoperative, and 1-month postoperative; 22 cases per timepoint, total n=66; χ²=62.824, p<0.01). Preoperative assessment revealed predominantly moderate-to-severe grades (grade 3.0: 59.09%; grade 2.0: 22.73%), with complete absence of grade 0.0 cases. Marked improvement was observed at 1-week post-intervention, with 59.09% achieving grade 0.0 and 40.91% presenting grade 1.0, while all higher grades (2.0 and 3.0) were completely resolved. By 1-month follow-up, the therapeutic outcomes further improved, with 95.45% of cases attaining grade 0.0 and only 4.55% remaining at grade 1.0. This temporally progressive improvement pattern, supported by robust statistical significance (p<0.01), not only confirms the rapid treatment efficacy (evident at 1-week evaluation) but also demonstrates sustained anatomical restoration (near-complete normalization at 1-month), providing objective evidence for clinical outcome assessment (**Table 2**).

**Table 1:** LIPCOF Grades: Pre- and Post-Surgery.

Case	Gender	Age	LIPCOF Grades			Postoperative Complications at 1 Month	Treatment Method
			Preoperative	Postoperative 1 Week	Postoperative 1 Month		
1	M	76	III	I	0		
2	F	76	III	I	0		
3	F	86	III	0	0		
4	M	72	III	0	0		
5	F	67	II	0	0		
6	F	70	III	0	0		
7	F	61	II	0	0		
8	M	66	III	0	0		
9	F	73	I	0	0		
10	M	56	I	0	0		
11	F	73	I	0	0		
12	F	73	III	0	0		
13	M	53	II	0	0		
14	M	71	I	0	0		
15	F	73	III	0	0		
16	F	70	III	I	I	Delayed resolution of edema	treatment with loteprednol etabonate eye drops (BID) and sodium hyaluronate eye drops (QID)
17	F	72	III	I	0		
18	F	85	II	I	0		
19	F	47	III	I	0		
20	F	47	II	I	0		
21	M	73	III	I	0		
22	M	73	III	I	0		

**Table 2:** Proportion of LIPCOF grades before and after surgery.

LIPCOF Grades	Group (%)			Total	χ2	*p*
	pre	post 1w	post 1m			
0.0	0 (0.00)	13 (59.09)	21 (95.45)	34 (51.52)	62.824	0.000**
1.0	4 (18.18)	9 (40.91)	1 (4.55)	14 (21.21)		
2.0	5 (22.73)	0 (0.00)	0 (0.00)	5 (7.58)		
3.0	13 (59.09)	0 (0.00)	0 (0.00)	13 (19.70)		
Total	22	22	22	66		

**p<0.01.

### 3.3 OSDI scores

Given the non-normal distribution of OSDI scores (Kolmogorov-Smirnov and Shapiro-Wilk tests, both p<0.01), nonparametric analyses were performed. The Kruskal-Wallis test revealed significant differences among the preoperative, 1-week postoperative, and 1-month postoperative groups (*H*=38.017, p<0.001) (**Table 3**). Dunn’s *post hoc* tests demonstrated: (1) no significant difference between preoperative (median=38.54, IQR=31.3-49.0) and 1-week postoperative groups (41.67, 32.3-50.5; p=0.922); (2) significantly lower scores in the 1-month postoperative group (16.67, 12.5-18.8) compared to both preoperative (p<0.001) and 1-week postoperative groups (p<0.001). The narrow IQR in the 1-month group indicates consistent symptom improvement after surgical intervention, while the wider variability in preoperative and 1-week groups reflects greater symptom severity. These findings confirm that surgical treatment leads to significant and progressive improvement in clinical symptoms, with optimal outcomes observed at 1-month follow-up (**Table 4**).

**Table 3:** OSDI scores before and after surgery.

	Median (IQR) of Groups (P25,P75)			*H*	*p*
	1.0 (*n*=22)	2.0 (*n*=22)	3.0 (*n*=22)		
OSDI	38.540 (31.3,49.0)	41.670 (32.3,50.5)	16.670 (12.5,18.8)	38.017	0.000**

**p<0.01; 1.0: Preoperative group; 2.0: 1-week postoperative group; 3.0: 1-month postoperative group.

**Table 4:** Intergroup differences in OSDI scores at three assessment timepoints.

	(I) Group	(J) Group	(I) Median	(J) Median	Mean Difference (I-J)	*p*
OSDI	1.0	2.0	38.540	41.670	-3.130	0.922
	1.0	3.0	38.540	16.670	21.870	0.000**
	2.0	3.0	41.670	16.670	25.000	0.000**

*p<0.05 **p<0.01; 1.0: Preoperative group; 2.0: 1-week postoperative group ; 3.0: 1-month postoperative group.

### 3.4 Wound healing

One week after surgery, examination with fluorescein staining using a slit lamp revealed that the areas of the conjunctiva that were touched and shrunk by the electrode tip did not absorb the stain, indicating complete healing (**Figure 3C**). Even so, there was still some staining in the incisions where the conjunctiva was clamped with the new conjunctival forceps, resected, cauterized, and sealed with electrocautery, suggesting incomplete healing (**Figure 3D**). Those incisions had a median healing rate of 75% at one week. By one month postoperatively, none of the incisions had the stain removed, indicating complete healing.

### 3.5 Postoperative observations and complications

All 22 eyes demonstrated expected postoperative inflammatory responses, including grade 1–2 bulbar conjunctival congestion and transient edema during the initial postoperative week (**Figure 3B**). These physiological healing responses resolved spontaneously in 21 eyes (95.5%) by the 1-month follow-up.

One patient (1/22, 4.5%) with preoperative LIPCOF grade 3 demonstrated delayed recovery, presenting with persistent conjunctival edema at 3 weeks postoperative and residual LIPCOF grade 1 at 1-month follow-up. This complication occurred despite initially prescribed postoperative medication (loteprednol etabonate eye drops BID and sodium hyaluronate eye drops QID), with confirmed non-compliance as the contributing factor. After therapeutic reinforcement including medication adherence education and adjusted anti-inflammatory regimen (increased loteprednol etabonate to TID for 2 weeks), complete anatomical resolution was achieved at the 6-week follow-up, with restoration of normal conjunctival architecture.

The overall surgical success rate was 95.5% (21/22) at 1-month follow-up, with no observed true surgical complications (including under correction/overcorrection, subconjunctival hemorrhage, symblepharon, fornix shortening, conjunctival scarring, or infection). All cases demonstrated either spontaneous resolution (21 eyes) or medication-responsive resolution (1 eye) of postoperative findings.

## 4 Discussion

Conjunctival laxity induces mechanical dysfunction, lacrimal pooling, and obstruction of the inferior punctum, resulting in abnormal tear film dynamics and dry eye syndrome in patient’s refractory to conservative management (5). Surgical intervention is required to restore normal bulbar conjunctival tension. In this study, sutureless conjunctivoplasty using novel specialized forceps combined with high-frequency electrocautery demonstrated significant clinical improvement, while effectively reducing operative time and eliminating postoperative suture removal requirements.

Conventional techniques for treating Cch entail conjunctival resection, in which the superfluous loose conjunctiva is surgically removed in the form of a crescent under a microscope. The incision is then sutured with absorbable sutures, which are taken out a week following the procedure. This approach of crescent-shaped conjunctival resection with absorbable sutures, are limited by prolonged operative time (≥15 minutes), visible scarring from large incisions (≥5 mm), and delayed recovery (>1 week post-suture removal) (9). More critically, precision in tissue excision remains challenging as it depends entirely on the surgeon’s intraoperative judgment, carrying inherent dual risks of both under correction and overcorrection. The positive aspect of our innovative surgical technique is that it solves the issue definitively, thus obviating the need for the application and removal of sutures (10).While Harley et al. (11) demonstrated that conjunctival excision combined with amniotic membrane transplantation (AMT) reduces fornix shortening risk, this technique retains suture-related morbidities (e.g., granuloma, infection). Similarly, electrocautery-based methods (12) and scleral fixation (13) fail to address the core limitations of residual laxity in moderate-to-severe Cch (grade II–III) and technical complexity, respectively.

This article presents a novel kind of conjunctival forceps made of titanium alloy that may maximally clamp extra lax conjunctival tissue and fix it without the assistance of a helper. After the resection, the incision was coagulated using high-frequency electrocautery. The average surgical time was (6.9 ± 1.5) minutes, representing a ​​42% reduction compared to traditional surgery, significantly minimizing intraoperative time and patient discomfort. Despite incomplete incision healing at 1 week, ​​OSDI scores showed no significant improvement​​ compared to preoperative levels, which is consistent with the expected delayed recovery after high-frequency coagulation. However, at ​​1-month follow-up​​, the incision had fully healed, and OSDI scores significantly decreased, indicating substantial relief from ocular discomfort and pain. Among our patients, 81.4% were preoperatively diagnosed with grades II and III Cch. Despite the presence of bulbar conjunctival edema one week after the operation, the severity of Cch in all patients was reduced to grade 0 or I. At One month after the operation, the conjunctival edema resolved, the incision healed, and 95.5% of the patients achieved grade 0. Thus, even those with grade II and III Cch can benefit from quick and convenient correction. One month after surgery, a patient (4.5%) continued to show grade I Cch due to persistent bulbar conjunctival edema, which resulted from non-adherence to the prescribed medication regimen.

High-frequency electrocautery, a staple in ophthalmology, is used for a variety of procedures, including electrolysis of trichiasis, removal of conjunctival tumors, and achievement of hemostasis in ocular plastic surgery. It causes less tissue damage because of its lower energy output as compared to an electrocoagulator. Using high-frequency electrocautery (with optimal settings: 40–70°C, <15 μm lateral thermal spread) for hemostasis, we achieved ​​superior safety and efficiency​​. Key precautions include maintaining a dry surgical field and performing excisions in eyelid-covered areas to prevent wound dehiscence (14, 15).

Although our technique achieved remarkable efficacy in managing Grades II–III Cch (95.5% success rate), its applicability to severe cicatricial Cch or cases with pronounced conjunctival scarring remains unverified. Furthermore, anatomical constraints—including shallow fornices or tight eyelid margins—may hinder optimal instrument maneuverability, necessitating individualized procedural adjustments.

In summary, our preliminary experience with this novel conjunctival forceps combined with high-frequency electrocautery demonstrates promising outcomes for sutureless correction of conjunctival relaxation, including reduced operative time and avoidance of suture-related complications. However, this study has several limitations: the small sample size (22 eyes), lack of a control group, and absence of formal cost-analysis preclude definitive claims about cost-effectiveness or superiority over standard techniques. While the procedure appeared safe in this cohort, larger comparative studies with long-term follow-up are needed to validate its efficacy, safety, and economic advantages. Despite these limitations, the technique’s simplicity and elimination of suture removal warrant further investigation as a potential alternative for select cases.

## Data Availability

The original contributions presented in the study are included in the article/**Supplementary Material**. Further inquiries can be directed to the corresponding author.

## References

[1] Yvon C Patel BC Malhotra R. Conjunctivochalasis. In: StatPearls. Treasure Island (FL): StatPearls Publishing (2023). 35015435

[2] Gan JY Li QS Zhang ZY Zhang W Zhang XR. The role of elastic fibers in pathogenesis of conjunctivochalasis. Int J Ophthalmol. (2017) 10:1465–73. doi: 10.18240/ijo.2017.09.21, PMID: 28944209 PMC5596235

[3] Ballesteros-Sánchez A Sánchez-González JM Borrone MA Borroni D Rocha-de-Lossada C. The influence of lid-parallel conjunctival folds and conjunctivochalasis on dry eye symptoms with and without contact lens wear: A review of the literature. Ophthalmol Ther. (2024) 13:651–70. doi: 10.1007/s40123-023-00877-9, PMID: 38217793 PMC10853109

[4] Balci O. Clinical characteristics of patients with conjunctivochalasis. Clin Ophthalmol (Auckland N.Z.). (2014) 8:1655–60. doi: 10.2147/OPTH.S61851, PMID: 25210435 PMC4155901

[5] Yang J Chandwani R Gopinatth V Boyce T Pflugfelder SC Huang D. Near-infrared laser thermal conjunctivoplasty. Sci Rep. (2018) 8:3863. doi: 10.1038/s41598-018-22204-0, PMID: 29497112 PMC5832782

[6] Marmalidou A Palioura S Dana R Kheirkhah A. Medical and surgical management of conjunctivochalasis. ocular surface. (2019) 17:393–9. doi: 10.1016/j.jtos.2019.04.008, PMID: 31009751

[7] Cheng AMS Mead OG Tighe S Tseng SCG. Fornix deepening reconstruction in conjunctivochalasis surgery. Taiwan J Ophthalmol. (2022) 13:49–54. doi: 10.4103/tjo.tjo_28_22, PMID: 37252158 PMC10220437

[8] Trivli A Dalianis G Terzidou C. A quick surgical treatment of conjunctivochalasis using radiofrequencies. Healthcare (Basel Switzerland). (2018) 6:14. doi: 10.3390/healthcare6010014, PMID: 29439532 PMC5872221

[9] Ucar F Unluzeybek M. Comparison of 2 different treatments for conjunctivochalasis: plasma-based conjunctivoplasty versus argon laser photocoagulation. Cornea. (2024) 43:1257–63. doi: 10.1097/ICO.0000000000003464, PMID: 38207054

[10] Xie J Zhang CX. An evaluation of partial conjunctival resection for conjunctivochalasis [in Chinese]. Chin J Optom Ophthalmol Vis Sci. (2020) 22(3):180–3.

[11] Harley U Salvi SM. Fibrin glue assisted amniotic membrane graft reconstruction of conjunctival fornix and canthus following excision of conjunctival tumors. Indian J Ophthalmol. (2022) 70:1033–6. doi: 10.4103/ijo.IJO_1704_21, PMID: 35225569 PMC9114583

[12] Çağlayan M Kösekahya P Gürdal C Saraç Ö. Comparison of electrocoagulation and conventional medical drops for treatment of conjunctivochalasis: short-term results. Turkish J Ophthalmol. (2018) 48:61–5. doi: 10.4274/tjo.35002, PMID: 29755817 PMC5938477

[13] Wang H Gao F Pan YZ. The treatment outcomes of crescent-shaped conjunctiva resection combined with conjunctiva sclera fixation for severe conjunctivochalasis. Eur Rev Med Pharmacol Sci. (2016) 20:3519–22., PMID: 27649649

[14] Ji YW Seong H Lee S Alotaibi MH Kim TI Lee HK. The correction of conjunctivochalasis using high-frequency radiowave electrosurgery improves dry eye disease. Sci Rep. (2021) 11:2551. doi: 10.1038/s41598-021-82088-5, PMID: 33510304 PMC7844232

[15] Kim B Lee Y Son HS Choi CY. Clinical outcomes of conjunctivochalasis treatment with a new ophthalmic radiofrequency device. BMC Ophthalmol. (2024) 24:302. doi: 10.1186/s12886-024-03499-2, PMID: 39039541 PMC11265150

