# Supplementary material for: Efficacy of a novel surgical approach: sutureless correction of conjunctivochalasis using new conjunctival forceps combined with high-frequency electrocautery
Source: Front Ophthalmol (Lausanne). 2025 Jul 11;5:1554316. doi: 10.3389/fopht.2025.1554316 (PMC12289505; doi:10.3389/fopht.2025.1554316)
Supplement: Supplementary file 1 [file Table1.docx]

Video 1. Conjunctivochalasis surgery using a new conjunctival forceps combined with high-frequency electrocautery.
